# Third-Stage Complications Among In Vitro Fertilization Pregnancies: An Observational Study

**DOI:** 10.7759/cureus.63038

**Published:** 2024-06-24

**Authors:** Reema T Magar, Parvathi Tejanaik, Haritha Sagili

**Affiliations:** 1 Obstetrics and Gynecology, Jawaharlal Institute of Postgraduate Medical Education & Research, Puducherry, IND

**Keywords:** in vitro fertilization (ivf), third stage, manual removal of placenta, retained placenta, postpartum hemorrhage

## Abstract

Objective: This study aimed to determine the third-stage complications and their risk factors in in vitro fertilization (IVF) pregnancies.

Methods: This prospective observational study was conducted from March 2022 to November 2023 at a tertiary care university hospital in South India. We included a total of 217 women following IVF conception, and details of the third-stage labor complications were documented and expressed as the frequency with percentage. The risk factors were analyzed using a logistic regression model.

Results: Among 217 participants, 51 (23.5%) had third-stage complications. Postpartum hemorrhage (PPH) was the most common, complicating 20% of the deliveries. Multiple gestations (adjusted odds ratio (aOR) 2.7, 95% confidence interval (CI) 1.03-7.46, p = 0.04), operative vaginal delivery (aOR 57, 95% CI 4.2-770, p = 0.002), and emergency cesarean section (aOR 14.8, 95% CI 1.3-160.5, p = 0.026) were the risk factors for PPH. Intrapartum infection was found to be associated with a risk for the retained placenta (aOR 8, 95% CI 1.37-46.4, p = 0.02) and adherent placenta (aOR 6.06, 95% CI 1.07-34.3, p = 0.04). Assisted reproductive technology (ART)-related factors were not found to be significantly associated with third-stage complications.

Conclusion: There is a risk of third-stage complications, especially postpartum hemorrhage, among IVF pregnancies. The type of embryo transfer was not associated with third-stage complications.

## Introduction

The third stage of labor begins from the delivery of the baby to the expulsion of the placenta and membranes. Active management of the third stage of labor (AMTSL) is recommended as a critical intervention for the prevention of postpartum hemorrhage (PPH) [1].

Assisted reproductive technology (ART) pregnancies have increased worldwide. It comprises 1% of all pregnancies in the United States and one-third of all multiple pregnancies [2]. The National ART and Surrogacy Registry (NARTSR) has recorded similar growth in ART clinics and treatment in India [3]. Although many studies, mostly retrospective, have shown that pregnancies following ART are associated with increased third-stage complications like a prolonged third stage, retained placenta, adherent placenta, manual removal of placenta, postpartum hemorrhage (PPH), blood transfusion, and intensive care unit (ICU) admission [2,4-10], it is unclear whether ART procedure, underlying infertility, or maternal antenatal factors are the reason behind these adverse third-stage outcomes. In vitro fertilization (IVF)-related factors like endometrium preparation and receptivity, ovarian stimulation, type of embryo (fresh/frozen), number of embryos transferred, and genetic alternation may also contribute to these adverse outcomes. Age, BMI, duration of infertility, and prior cesarean section are some of the maternal factors that can also affect pregnancy and third-stage outcomes [4].

The primary factor responsible is probably embryonic and not endometrial. The Rohr fibrinoid layer's thickness and decidual loss rate in frozen embryo transfer (FET) cycles were positively correlated with the amount of bleeding following deliveries. Differential gene expression of CACNA1I, responsible for regulating uterine radial arteries vasoconstriction, is critical for normal placentation, hormone secretion, and fetal development. In addition, calcium channels are essential for effective uterine contraction; in the absence of effective contraction, the atonic uterus can bleed excessively, resulting in PPH. Decreased expression of CACNA1I is noted in IVF pregnancies than in spontaneous conceptions [9].

There is a lack of prospective data concerning third-stage outcomes specific to ART pregnancies. We aimed to determine the third-stage complications and the factors associated with these complications following IVF.

## Materials and methods

This prospective observational study was conducted in the Department of Obstetrics & Gynecology, Women and Children Hospital of Jawaharlal Institute of Postgraduate Medical Education & Research, Puducherry, India, from March 2022 to November 2023. The Institutional Ethics Committee approved the study protocol (JIP/IEC/2022/014). Annually, approximately 15,000 deliveries (vaginal and cesarean) are conducted in our center. After taking informed written consent, we enrolled all pregnant women with gestational age ≥28 weeks conceived following IVF. Women with diagnosed uterine anomalies and a previous history of manual removal of the placenta were excluded.

A pre-designed proforma was used to collect maternal sociodemographic factors, menstrual/marital/obstetric (present and past) details, and details of ART, i.e., indication for IVF, ovulation induction protocol, and details of embryo transfer. Gestational age was calculated from the day of embryo transfer. Antenatal records were checked for any pre-existing medical or surgical comorbid conditions, and ultrasonography findings were reviewed for the number of fetuses, liquor volume, fetal anomalies, location, and adherence of the placenta. All study participants received AMTSL, i.e., injection oxytocin 10 IU intramuscular within one minute of delivery, followed by delayed cord clamping (two to three minutes) and placental delivery by control cord traction.

Details of third-stage complications and management were documented. The third-stage complications studied were prolonged third stage (>30 minutes), retained placenta (placenta not expelled within 30 minutes of childbirth), adherent placenta, manual removal of placenta (MRP), PPH (blood loss of ≥500 mL within 24 hours following vaginal delivery/≥1000 ml following cesarean delivery), peripartum hysterectomy, need for blood products, and admission to intensive care unit (ICU). Blood loss following vaginal delivery was measured using a disposable calibrated obstetric drape and weighing the soaked vaginal pads over 24 hours. In women who underwent cesarean delivery, intraoperative blood loss was calculated by weighing the soaked mop and the volume of blood in the suction bottle following the suctioning of amniotic fluid and the delivery of the baby by connecting to a separate suction apparatus. The soaked mop and vaginal pads were weighed by a digital scale and recorded in grams. The difference in weight between a dry and soaked mop and vaginal pads was estimated, and the blood loss weight measured in grams was converted to milliliters by dividing the weight by 1.06 (blood density in grams per milliliter).

The sample size was calculated using OpenEpi version 3.01. Based on a previous study [2], we considered manual removal of the placenta as a third-stage complication in ART pregnancy following frozen embryo transfer to be 17%, the sample size calculated as 217 with 5% absolute precision, and a confidence level of 95%.

Statistical analysis was performed using Stata statistical software (release 14, 2015, StataCorp LLC. College Station, TX). Continuous variables were expressed as mean with standard deviation or median with interquartile range depending upon the normality distribution. The Kolmogorov-Smirnov test was used to assess the normality. Categorical variables were expressed as the frequency with percentage. The outcomes were expressed as frequency with percentages. Multivariate logistic regression analysis was performed to study the association of various risk factors with third-stage complications. To adjust for confounders, linear/logistic regression analysis was done. A p-value <0.05 was considered significant.

## Results

Demographic and pregnancy characteristics

During the study period, 217 eligible participants were recruited and followed up till discharge from the hospital. Demographic and maternal characteristics are presented in Table 1.

**Table 1:** Demographic and pregnancy characteristics of the study participants Abbreviations: BMI: body mass index; LSCS: lower-segment cesarean section; ART: assisted reproductive technology; GnRH: gonadotrophin-releasing hormone, ET: embryo transfer; OVD: operative vaginal delivery ^a^ Values are given as number (percentage) or mean ± standard deviation.

Variables	Frequency (n = 217) (%)^a^
Age (years)	33.6 ± 5.9
BMI (kg/m^2^)	31.9 ± 5.23
Gestational age	35.6 ± 2.56
Gravidity	
Primigravida	147 (67.74)
Multigravida	70 (32.26)
Plurality	
Singleton	116 (53.46)
Multiple	101 (46.54)
Co-existing conditions	
Hypertensive disorders	139 (64.06)
Diabetes mellitus	88 (40.05)
Hypothyroidism	41 (18.8)
Anemia	81 (37)
Prior surgeries	76 (35.02)
Infertility causal factors	
Male factor	90 (42)
Female factor	98 (45)
Combined factors	29 (13)
ART details	
GnRH antagonist	124 (57.24)
GnRH agonist	17 (7.84)
Frozen ET	207 (95.4)
Fresh ET	10 (4.6)
Onset of labor	
Spontaneous	57 (26)
Induced	48 (22)
Not in Labor	112 (52)
Mode of delivery	
Vaginal delivery	25 (11)
OVD	18 (9)
Emergency LSCS	104 (48)
Elective LSCS	70 (32)

The mean age of the study population was 33 ± 5.9 years, and 53% were more than 30 years of age. The mean BMI was 31.91 ± 5.23 kg/m^2^ and more than three-fourths of them were obese. Two-thirds of them are primigravida and had singleton pregnancies following IVF. Around 103 (47.5%) participants had multiple medical comorbid conditions. Two-thirds had hypertensive disorder, and 40% had diabetes. Among hypertensive disorders, the proportion of preeclampsia was the highest (56%). One-third of the women had mild anemia, and none had severe anemia.

One-third of the participants had a history of previous abdominal or pelvic surgery, and the most common surgical procedure performed was ovarian drilling for polycystic ovarian syndrome (PCOS). The mean gestational age at delivery was 35.6 ± 2.56 weeks, the earliest at 28 weeks, and the term at 40 weeks. A total of 174 (80%) women delivered by cesarean delivery (emergency and elective). The maternal request was the most common indication.

Two-thirds of the participants were suffering from primary infertility, with a mean duration of five to 10 years. Both male and female factors were found to be in equal proportion, around 42-45% contributing to their infertility. More than half of the participants had ovarian stimulation with GnRH antagonist protocol, and the majority had conception following frozen embryo transfer (95.4%).

Third-stage complications

Among 217 participants, 51 participants (23.5%) had third-stage complications. Details of the third-stage complications are shown in Table 2.

**Table 2:** Details of third-stage complications ICU: intensive care unit

Complications	Frequency (n = 217)	Percentage
Retained placenta	12	5.53
Manual removal of placenta	16	7.37
Adherent placenta	15	6.9
Focal adherence	3	
Diffuse adherence	12	
Postpartum hemorrhage	45	20.74
Prolonged third stage	16	7.37
Need for blood transfusions	33	15.21
Admission to ICU	37	17.05

The mean duration for the third stage was 10.8 ± 8.25 minutes. Postpartum hemorrhage was the most common third-stage complication noted in 45 (20.74%) of the participants, contributing to 88% of third-stage complications. Multiple gestation was an independent variable associated with the risk of PPH (aOR 2.71, 95%CI 1.03-7.46, p = 0.04). Other significant adverse third-stage outcomes were ICU admission (17%) and the need for blood transfusions (15%) secondary to PPH. The retained placenta was encountered in 12 (5.5%) participants; the placenta was separated and retained in the uterine cavity. In 11 of them, the placenta was delivered with oxytocin infusion (20 IU), and one participant underwent manual removal of the placenta. In participants with adherent placenta (focal and diffuse), the placenta was removed manually. In cases of diffuse adherence, umbilical vein injection of oxytocin (10 IU) was administered before attempting manual removal of the placenta. None of them had surgical intervention. There was no case of peripartum hysterectomy.

Table 3 shows a multivariate logistic regression analysis for maternal risk factors associated with third-stage complications. Intrapartum infection increased the risk of retained placenta (aOR 8, 95%CI 1.37-46.4, p = 0.02) and adherent placenta (aOR 6.06, 95% CI 1.07-34.3, p = 0.04).

**Table 3:** Multivariate logistic regression analysis of variables associated with third-stage complications OVD: operative vaginal delivery

S. no.	Variables	PPH		Retained placenta		Adherent placenta		Need for blood transfusion	
		Adjusted OR (95%CI)	P value	Adjusted OR (95%CI)	P Value	Adjusted OR (95%CI)	P value	Adjusted OR (95%CI)	P value
1	Multiple gestations	2.77 (1.03-7.46)	0.043	0.55 (0.16-1.9)	0.35	0.55 (0.18-1.67)	0.29	2.65 (0.99-7.09)	0.052
2	Mean GA at delivery	0.84 (0.69-1.02)	0.08	0.94 (0.76-1.18)	0.64	0.94 (0.77-1.15)	0.59	0.77 (0.64-0.93)	0.008
3	Mode of delivery								
	OVD	57 (4.2-770)	0.002	1.5 (0.08-25.7	0.78	0.59 (0.07-4.69)	0.63	2.08 (0.49-8.81)	0.316
	Elective LSCS	6.46 (0.46-90.67)	0.166	0.7 (0.06-8.14)	0.7	0.57 (0.17-1.91)	0.36	0.52 (0.18-1.46)	0.218
	Emergency LSCS	14.87 (1.3-160.5)	0.026	1.9 (0.23-16.5)	0.52	1	-	1	-
4	Intrapartum infection	2.2 (0.32-15.05)	0.41	8 (1.37-46.4)	0.020	6.06 (1.07-34.3)	0.042	2.3 (0.42-12.4)	0.33
5	Mean hemoglobin	0.70 (0.50-0.98)	0.03	0.84 (0.51-1.36)	0.48	0.86 (0.55-1.33)	0.50	0.81 (0.59-1.11)	0.20

ART-related factors like ovarian stimulation protocol did not reveal a significant risk of third-stage complications. The association of the type of ET to third-stage complications could not be assessed due to the lack of participants in the fresh ET group (only 4.6% had a fresh ET). History of cesarean delivery was found to increase the risk of retained placenta (aOR 2.48, p = 0.4), adherent placenta (aOR 1.76, p = 0.61), manual removal of placenta (aOR 1.6, p = 0.6), and postpartum hemorrhage (aOR 1.3, p = 0.75); however, it was statistically insignificant.

## Discussion

Advances in ART in the past decades have been a boon for couples suffering from infertility. With rising incidences of infertility, ART services have increased significantly worldwide and also in low- and middle-income countries like India [3]. Conceptions following IVF are considered high-risk pregnancies, mostly due to underlying maternal factors like age, high BMI, associated comorbid conditions, and increased risk of multiple pregnancies. In our study, the mean maternal age was 33 years, and more than half of them developed preeclampsia, similar to the studies by Kazuki Saito et al. and Ze Wang et al. [7,11,12]. The increased risk of pre-eclampsia has been speculated to be associated with endometrium preparation methods and the absence of corpus luteum in IVF pregnancies.

The majority of the conceptions in our study followed frozen ET (95%), which agrees with the worldwide trend of the popularity of frozen ET in comparison to fresh ET, mostly due to its convenience of postponing cycles to achieve optimal prenatal conditions. Autologous gametes were used mostly in 59% of our participants, followed by donor oocytes in 25%. Autologous gametes are involved with higher pregnancy rates [7]. ET has been found to be an independent risk factor for PPH [7,13-15]. Our study showed no statistically significant relation between type of embryo transfer, autologous vs donor embryo, co-existing medical conditions, age, and BMI on adverse third-stage outcomes.

In our study, 23.5% had third-stage complications. PPH was the most common complication in 20% of our study participants. The incidence of PPH in IVF pregnancy is found to be four times as high compared to the natural conception (20% vs. 5%); similar observations were seen in other studies [6,9,10,13]. Maternal age was associated with an increased risk of PPH and retained placenta [4,7]. Overall, in our study, maternal age was not associated with the risk of third-stage complications. Some of the previous studies showed an increased risk of PPH with the induction of labor. However, no statistically significant relation was found between labor induction with retained placenta, adherent placenta, prolonged third stage, and manual removal of placenta in our study, similar to the study by Avital et al. (OR 1.11, p = 0.75) [10].

Chorioamnionitis has been reported as an independent risk for manual removal of the placenta [5]. Our study results also concurred that intrapartum infection increased the risk of retained placenta, adherent placenta, and manual removal of placenta.

The study's strength was its prospective design, with a robust collection of all the maternal details related to ART and close follow-up till discharge for all the postpartum complications. The main limitation was the smaller sample size and lack of a comparative group. There were insufficient participants in the fresh ET group for comparison with frozen ET among IVF pregnancies for third-stage complications. Moreover, we did not have a follow-up after discharge from the facility to assess the secondary PPH and puerperal sepsis. We did not assess for perinatal outcomes.

As most of the women undergoing IVF are elderly and nulliparous with prolonged periods of infertility, obstetricians need to offer counselling not only on antepartum complications but also on birth plans and postpartum complications associated with these pregnancies.

## Conclusions

IVF pregnancy is a high-risk condition, not only in the antepartum but also in the intrapartum and postpartum periods. In our study, third-stage complications, namely, postpartum hemorrhage, retained placenta, manual removal of placenta, prolonged third-stage, adherent placenta, and need for blood transfusion, were observed in deliveries following IVF conception. ART-related factors did not reveal a significant risk of third-stage complications.
